# Does Learning to Read Improve Intelligence? A Longitudinal Multivariate Analysis in Identical Twins From Age 7 to 16

**DOI:** 10.1111/cdev.12272

**Published:** 2014-07-24

**Authors:** Stuart J Ritchie, Timothy C Bates, Robert Plomin

**Affiliations:** The University of Edinburgh; King's College London

## Abstract

Evidence from twin studies points to substantial environmental influences on intelligence, but the specifics of this influence are unclear. This study examined one developmental process that potentially causes intelligence differences: learning to read. In 1,890 twin pairs tested at 7, 9, 10, 12, and 16 years, a cross-lagged monozygotic-differences design was used to test for associations of earlier within-pair reading ability differences with subsequent intelligence differences. The results showed several such associations, which were not explained by differences in reading exposure and were not restricted to verbal cognitive domains. The study highlights the potentially important influence of reading ability, driven by the nonshared environment, on intellectual development and raises theoretical questions about the mechanism of this influence.

Investigation of the causes of intelligence differences has shown substantial roles for both genetic influences and environmental effects (Deary, 2012; Plomin, Haworth, Meaburn, Price, & Davis, 2013). The specific environmental mechanisms responsible for a portion of the variance in intelligence, however, are unclear. Here, we examine the effects of one potential environmental influence on intelligence: learning to read in childhood. Using a longitudinal monozygotic (MZ) twin differences design, we test whether twins who—for purely environmental reasons—acquire better reading skills than their cotwin show improvements in intelligence, and whether these associations are found across five waves of testing. Such a finding would have implications for educational interventions, and may also provide a partial answer to the important question of why children within a family have very different intelligence test scores, despite sharing factors such as genes, parental education, parental personality, and socioeconomic status (Plomin, 2011; Plomin & Daniels, 1987).

The ability to read and comprehend text is undoubtedly important in modern society, and reading ability has been associated with improved health (Baker, Parker, Williams, Clark, & Nurss, 1997), education (Duncan et al., 2007; McGee, Prior, Willams, Smart, & Sanson, 2002), socioeconomic status (Ritchie & Bates, 2013), and creativity (Ritchie, Luciano, Hansell, Wright, & Bates, 2013; Wang, 2012). Whereas reading may directly improve these variables—for instance, the ability to extract information from texts is of great importance in gaining educational qualifications—an additional mechanism for these associations may be that reading has a causal effect on more general cognitive abilities that are themselves associated with better life-course outcomes (e.g., Gottfredson, 1997). In other words, reading may, over time, improve general intelligence. Given evidence that both reading ability (Torgesen, 2005) and interest (Wigfield, Guthrie, Tonks, & Perencevich, 2004) are amenable to intervention, findings showing that improvement of these factors could also boost the development of intelligence are of clear practical interest for educators.

Several studies have shown that measures of engagement in free reading (measured using Author Recognition Test scores) are predictive of subsequent verbal ability, even controlling for initial verbal ability test scores. For instance, in a study of 147 fourth- to sixth-grade children (9–12 years old), Echols, West, Stanovich, and Zehr (1996) found associations of print exposure with reading comprehension, receptive and sight vocabulary, and general knowledge 2 years later, controlling for initial scores in these domains (see also Cain & Oakhill, 2011; Cunningham & Stanovich, 1991). In a review article, Cunningham and Stanovich (1998) conclude that “those who read a lot will enhance their verbal intelligence; that is, reading will make them smarter” (p. 7).

Whereas previous studies are positive about the potential effects of reading on other cognitive abilities, there are multiple interpretations that are compatible with the data. Possible explanations include a shared genetic basis for reading and cognition (as shown by, e.g., Harlaar, Hayiou-Thomas, & Plomin, 2005; Haworth, Meaburn, Harlaar, & Plomin, 2007), or influences of traits that themselves underpin reading, such as orthographic representation, on the development of verbal cognitive ability (Martin et al., 2009). Finally, engagement in free reading may reflect unmeasured traits related to interests that favor general knowledge, such as openness to experience, rather than reading skill per se.

For these reasons, we investigated the influence of reading ability—in addition to reading interest—on general intelligence, and tested our hypotheses using a longitudinal MZ twin differences design, as illustrated in Figure1 (see Vitaro, Brendgen, & Arseneault, 2009, for discussion of the logic of the design; see Burt, McGue, & Iacono, 2009; Spanos, Klump, Burt, McGue, & Iacono, 2010, for examples). This model controls for genetic and shared environmental effects (since MZ twins are genetically identical, and in this sample the twins were brought up in the same families), covariance between traits at each age (paths A and F in Figure1; this controls for potential influences that improve both reading and intelligence for one twin of a pair, such as an effective teacher), and the stability of traits across time (paths B and E), while allowing a test of our primary hypothesis that reading differences—in both ability and exposure—would be associated with later intelligence differences (path D). If paths of this nature were found, they would point toward a causal effect of reading on cognitive ability.

**Figure 1:**
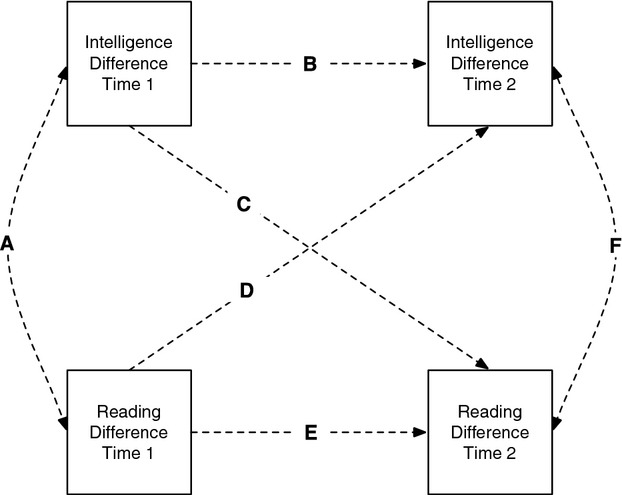
Cross-lagged longitudinal monozygotic twin difference model at two ages. All variables are intrapair difference scores. Significant values on the paths would indicate the following: A, covariance between intelligence and reading at Time 1; B, within-trait, across-time association for intelligence; C, between-trait, across-time intelligence–reading association; D, between-trait, across-time reading–intelligence association; E, within-trait, across-time association for reading; F, covariance between intelligence and reading at Time 2.

Our initial model used a measure of general intelligence (a composite made from a variety of tests at each age). This allowed us to go beyond the previous results (reviewed by Cunningham & Stanovich, 1998), which have focused on the more theoretically obvious links between the highly verbal skill of reading and verbal intelligence measures, and test for more wide-ranging effects of reading. It may be, for instance, that skills gained while learning to read are also of use in nonverbal intelligence tests, such as matrix reasoning. In addition to modeling reading and general intelligence, we tested for differential links to verbal and nonverbal subtests, to build a more detailed picture of the effects of reading on cognitive abilities.

At a mechanistic level, there are also reasons to suggest that enhanced general knowledge and cognitive ability may in turn enhance reading skill (i.e., the converse relation to that in the main hypothesis). Both dual-route (Coltheart, 2006) and connectionist (Seidenberg, 2012) models of reading implicate semantic knowledge in the reading process, via word representation activation. Moreover, domain knowledge has been shown to enhance reading and reading comprehension: Recht and Leslie (1988) found that prior domain knowledge enhanced reading skill. Our model also allowed us to test for these associations (path C in Figure1).

## Hypotheses

Our main hypothesis focused on the links between reading ability and intelligence; concurrent measures of these two abilities were available in our sample at ages 7, 9, 10, 12, and 16 years. If learning to read improves general intelligence, twins with higher reading scores than their co-twins at earlier ages should also have higher subsequent intelligence scores than their cotwins, controlling for earlier intelligence scores in the cross-lagged model. Significant paths of the type marked D in Figure1 at any point in the model would support this hypothesis. Simultaneously, we tested the converse hypothesis—that higher general intelligence aids in learning to read controlling for earlier reading ability—by assessing the significance of the paths marked C in Figure1.

The second model was constructed with an additional measure of reading exposure, which was available at ages 10 and 12; this model allowed us to test for intelligence-boosting effects of exposure while controlling for ability, and vice versa. If reading exposure improves intelligence above and beyond reading ability, we would expect to see significant paths from exposure differences to intelligence differences in this model.

Finally, a third model, with data from all five waves, assessed the links between reading and verbal and nonverbal intelligence measures separately. If reading operates only via verbal intelligence tests, such as vocabulary, to improve general intelligence, we should not expect to see any paths from earlier reading differences to later nonverbal intelligence differences in this model. However, if these paths were significant, this would be consistent with a positive effect of reading on a wider range of cognitive tasks than has thus far been considered.

## Method

### Participants

Participants were monozygotic twin pairs enrolled in the Twins Early Development Study (TEDS; Haworth, Davis, & Plomin, 2013), an ongoing longitudinal study of twins born in England and Wales between January 1994 and December 1996. The sample has been shown to be representative of the UK population of families of young children (Kovas, Haworth, Dale, & Plomin, 2007). Originally, the sample included data from 8,163 families; to date, participants have been followed up at 11 subsequent testing waves and data from 5 of these (ages 7, 9, 10, 12, and 16) are included in the present study. Each twin pair was raised in the same family. At age 7, data were available for 1,890 monozygotic twin pairs (1,001 female–female pairs) and at age 16, 1,258 pairs (635 female–female). Valid sample sizes for each measure at each age are shown in Table1. Zygosity was recorded by parents and shown by DNA analysis to have an accuracy rate at or above 95% (Price et al., 2000).

**Table 1:** Sample Sizes, Mean Scores, and Standard Deviations on Individual Reading and Intelligence Measures for All Participants

Age (years)	Test type	Measure (max. score)	*n*	*M*	*SD*
7	Reading	TOWRE (139)	3,760	58.80	27.59
		Teacher-rated reading (5)	4,336	2.15	.69
	IQ	Vocabulary (36)	3,797	13.00	4.24
		Similarities (22)	3,792	6.22	2.95
		Picture completion (21)	3,810	10.63	3.17
		Conceptual grouping (12)	3,810	7.93	2.43
9	Reading	Teacher-rated reading (5)	2,062	3.07	.77
	IQ	Vocabulary (40)	2,469	26.53	5.93
		Information (18)	2,446	11.74	2.79
		Figure classification (24)	2,461	18.13	4.38
		Figure analogies (23)	2,389	15.81	6.19
10	Reading	PIAT (82)	2,215	45.44	13.65
		Teacher-rated reading (5)	2,144	3.47	.83
		Author recognition test (−21 to 21)	1,911	7.32	3.83
	IQ	Vocabulary (60)	1,958	36.00	11.68
		Information (30)	1,940	19.68	4.60
		Picture completion (30)	1,945	19.13	4.22
		Raven's (60)	1,985	36.99	8.86
12	Reading	PIAT (82)	3,959	56.66	11.13
		GOAL (36)	3,887	22.84	6.66
		WJ–RF (100)	3,867	57.43	13.44
		TOWRE (167)	3,384	112.77	21.31
		Teacher-rated reading (9)	2,880	4.40	.95
		Author recognition test (−21 to 21)	3,287	8.77	3.84
	IQ	Vocabulary (60)	3,331	38.81	10.58
		Information (30)	3,535	21.01	4.25
		Picture completion (30)	3,247	19.69	4.01
		Raven's (24)	3,438	10.61	3.48
16	Reading	Passages (26)	1,598	17.04	3.99
		WJ–RF (98)	1,911	63.13	13.52
	IQ	Vocabulary (33)	2,006	15.11	4.21
		Raven's (30)	1,843	13.86	3.70

Note. *n* refers to individuals. All scores in this table uncorrected for age and sex. IQ = intelligence; TOWRE = Test of Word Reading Efficiency; PIAT = Peabody Individual Achievement Test; WJ–RF = Woodcock–Johnson Reading Fluency (yes/no); Raven's = Raven's Progressive Matrices.

### Measures

Details of the reading and intelligence measures in this sample have been published previously for ages 7, 9, and 10 (Kovas et al., 2007) and age 12 (Haworth et al., 2007). All measures described below were approximately normally distributed in both their raw and composite forms.

#### Reading Ability

Reading was measured at age 7 using the Test of Word Reading Efficiency (TOWRE; Torgesen, Wagner, & Rashotte, 1999), which assesses accuracy and fluency of reading both words and nonwords. This test was administered by telephone. In addition, reading was assessed at this age as UK National Curriculum (NC) achievement level, as recorded by each twin's classroom teacher. Teachers provided a reading score for each twin on a 5-point scale: 1 = *achievement well below the expected standard for the child's age*, 2 = *below the expected standard*, 3 = *at the expected standard*, 4 = *above the expected standard*, 5 = *exceptional achievement*. Previous TEDS studies have shown that these ratings correlate strongly with objective tests (e.g., Dale, Harlaar, & Plomin, 2005).

At age 9, reading was again assessed by teachers as each twin's UK NC level. At age 10, reading was assessed both as the UK NC level and on the reading comprehension subtest of the Peabody Individual Achievement Test (PIAT; Markwardt, 1997). At age 12, five measures of reading ability were completed: first, the PIAT; second, the GOAL Formative Assessment in Literacy for Key Stage 3, a UK curriculum-linked comprehension test (GOAL, 2002); third, an adaptation of the Woodcock–Johnson III Reading Fluency Test (WJ–RF; Woodcock, McGrew, & Mather, 2001); fourth, the TOWRE; and fifth, the UK NC level, at this age rated from 1 to 9. The first three tests were administered over the Internet (Haworth et al., 2007); the TOWRE was again administered by telephone.

At age 16, participants completed two tests of reading comprehension, also administered online: first, a further adaptation of the WJ–RF, with more difficult (and thus age-appropriate) items; second, the “Passages” test (designed specifically for TEDS), which involved reading two approximately 500-word passages and answering a series of 13 multiple-choice comprehension questions on each.

Where multiple reading measures were available at one age (i.e., all ages except 9), the *z* scores from each measure were summed, corrected for age and sex, and standardized before being used in the model. Tables S1 and S2 in the online Supporting Information show the correlations of these measures prior to this process; the reading variables correlated from *r*s =* *.28 to .69, and the intelligence variables from .09 to .54 (all *p*s < .001).

#### Reading Exposure

An online version of the Author Recognition Test (ART; Stanovich & West, 1989) was used to measure reading interest at ages 10 and 12. This involved a list of 42 names, half of which were names of popular children's authors and half of which were foils. Participants were asked to click on the names they thought were real author names and could click as many or as few as they wished. The list of authors was broadly the same in both tests, with three author names being replaced for the age 12 administration. The test was scored by subtracting the number of foils clicked from the number of real authors clicked (thus, the score potentially ranged from −21 to 21). Genetic and environmental relations between the ART and reading ability in this sample were analyzed and discussed by Harlaar, Dale, and Plomin (2007) and Harlaar, Trzaskowski, Dale, and Plomin (2014). ART scores were corrected for age and sex before inclusion in the analysis.

#### Intelligence

At age 7, intelligence was assessed using three subscales from the Wechsler Intelligence Scale for Children, Third Edition (WISC–III–UK; Wechsler, 1992)—Vocabulary, Similarities, and Picture Completion—as well as the Conceptual Grouping subtest from the McCarthy Scales of Children's Abilities (MSCA; McCarthy, 1972). The former two tests measure verbal intelligence, while the latter two measure nonverbal intelligence. These tests were administered individually by telephone, using a booklet mailed to the twins' home prior to testing (Petrill, Rempell, Dale, Oliver, & Plomin, 2002).

At age 9, intelligence was measured using four tests administered via a booklet completed by each twin at home. The four tests were the Vocabulary and Information tests, both multiple choice, from the WISC–III–PI (Kaplan, Fein, Kramer, Delis, & Morris, 1999) to assess verbal ability, and the Figure Classification and Figure Analogies subtests from the Cognitive Abilities Test 3 (CAT3; Smith, Fernandes, & Strand, 2001) to assess nonverbal ability. At ages 10 and 12, intelligence was assessed using Internet-based tests. Each twin logged on to the TEDS website and completed two multiple-choice tests of verbal ability, which were the Vocabulary and Information WISC–III–PI subtests and two tests of nonverbal ability, namely, the Picture Completion WISC–III–UK subtest and Raven's Standard Progressive Matrices (Raven, Court, & Raven, 1996). The tests used at age 12 included more difficult items than those used at age 10.

At age 16, intelligence was also measured using Internet tests, this time using one verbal ability test, the multiple-choice Mill Hill Vocabulary Scale (Raven et al., 1996) and one nonverbal ability test, Raven's Standard Progressive Matrices.

As with the reading measures, the intelligence test *z* scores recorded at each age were summed to produce a general intelligence score, then corrected for age and sex and standardized.

### Statistical Analyses

We entered intrapair differences in each measure (calculated by subtracting the score of the first randomly selected twin in each pair from that of the second) into cross-lagged longitudinal MZ difference models (Figure1), estimated using OpenMx (Boker et al., 2011) in the *R* environment, and using full information maximum likelihood estimation to handle missing data. Saturated structural equation models containing all possible paths were first estimated, then paths were set to zero (dropped) in several steps, outlined in the next section. This approach is necessarily exploratory, but reflects the lack of previous research into this question using measures of reading ability (rather than exposure), or using this structural equation modeling approach.

## Results

Descriptive statistics are shown for each individual reading and intelligence test in Table1, and three correlation matrices are provided in the online Supporting Information for the correlations of each reading test prior to summing into general scores (Table S1), the same for each intelligence test (Table S2), the correlations of the summed scores for both reading and intelligence (Table S3), and the correlations of the difference scores (Table S4).

**Figure 2:**
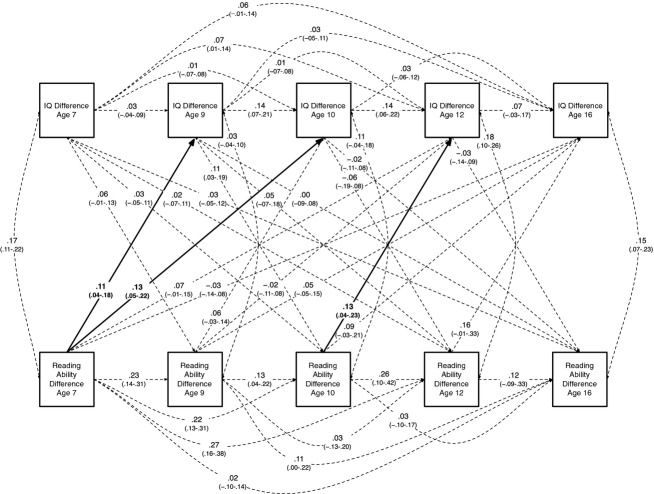
Saturated cross-lagged monozygotic twin difference model of the development of reading ability and general intelligence from ages 7 to 16. Values are standardized path coefficients; 95% confidence intervals are shown in parentheses. Bold paths indicate associations of earlier reading ability differences with later intelligence differences where 95% confidence intervals do not cross zero. IQ = general intelligence.

Our first model included reading ability and intelligence differences across all five ages, and the saturated model is shown in full, with confidence intervals around each path estimate, in Figure2. As highlighted with bold lines and type, in the saturated model three paths between reading and intelligence had confidence intervals that did not cross zero, indicating that they were statistically significant at *p* < .05, and supporting our main hypothesis that earlier reading differences are positively associated with subsequent intelligence differences.

Many of the paths in the saturated model had 95% confidence intervals that crossed zero, indicating they could be dropped with no significant decrement in fit. Since dropping them from the model could alter the weights of other paths, we tested whether our theoretically relevant paths were still significant after a process of path dropping. We dropped the paths according to two rules, chosen based on our saturated model, which indicated that chronologically later paths would tend to have smaller weights and thus be easier to drop from the model. The first rule was to begin by dropping these paths (e.g., between reading differences at ages 12 and 16). The second rule was to drop adjacent paths (e.g., between reading differences at ages 10 and 12) before longer term paths (e.g., between reading differences at ages 10 and 16).

We started by testing the within-trait (autoregressive) paths for reading (path E in Figure1, now extended across all five testing waves). After dropping the first path, the resulting model was compared to the saturated model. If this did not result in a significant change in model fit, the path was not reinstated, and the next path was dropped and tested, with the comparator being the most recent new model. If dropping the path resulted in a significant change in model fit, the path was deemed significant and retained for the final model. We then carried out the same process for the four remaining sets of paths in the following order: within-trait intelligence differences (path B in Figure1, again, across all five waves), cross-lagged associations between reading and intelligence (path D); cross-lagged associations between intelligence and reading (path C), and, finally, contemporaneous covariances between reading and intelligence (paths A and F).

**Figure 3:**
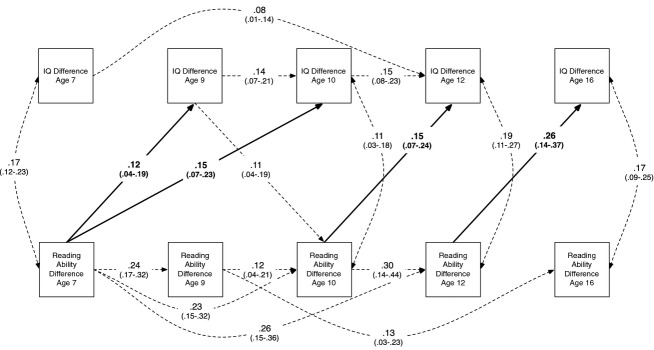
Reduced model, showing only significant paths, for cross-lagged monozygotic twin differences analysis of reading ability and intelligence (see saturated model in Figure2). Nonsignificant paths removed as per the path-dropping process outlined in the Results section; 95% confidence intervals for each path shown in parentheses; bold paths indicate significant associations of earlier reading differences with subsequent intelligence differences. IQ = general intelligence.

The final, reduced model after completion of this process, showing only the significant paths, is shown in Figure3. For within-trait reading difference associations, we could drop four paths without significant loss of fit (all resulting changes in fit are shown in Table2). For within-trait intelligence difference associations, we could drop a further seven paths. Six cross-lagged paths between intelligence and reading were dropped, as were nine cross-lagged paths between reading and intelligence. Finally, one of the five reading–intelligence covariance paths could be dropped. Overall, then, 27 paths were dropped without significant loss of fit (*p *=* *.42), and the final model had excellent fit to the data, χ^2^(27) = 27.84; root mean square error of approximation (RMSEA) = .003, comparative fit index (CFI) = .997, Tucker–Lewis index (TLI) = .996.

**Table 2:** Changes in Model Fit After Dropping Individual Paths via the Path-Dropping Process for the Model Shown in Figure 2, Resulting in the Model Shown in Figure 3

Path set	Change in fit after dropping paths		
	ΔAIC	Cumulative no. paths dropped (Δ*df*)	*p*
Reading: within-trait	−3.91	4	.39
IQ: within-trait	−9.76	11	.35
Reading → IQ: cross-lagged	−12.57	17	.21
IQ → reading: cross-lagged	−24.89	26	.40
IQ ↔ reading: covariance	−26.16	27	.42

Note. The saturated model included 45 paths; the final model included 18.

AIC = Akaike information criterion; IQ = intelligence; Reading = reading ability. Reference model AIC = 5905.05 on 27 *df*.

To test the robustness of our results, we attempted four alternative model-fitting processes. The first two of these examined whether our particular path-dropping process introduced any bias. First, we dropped the path sets in the reverse order to that described earlier, beginning with the contemporaneous covariances and ending with the within-trait reading paths. Second, we began with a base model specifying only within-trait and cross-lagged paths to adjacent ages (e.g., from ages 7 to 9), added any other paths that produced significant improvements in model fit (these paths were added in chronological order from the following sets: within-trait reading paths, within-trait intelligence paths, cross-lagged reading-to-intelligence paths, cross-lagged intelligence-to-reading paths). Whereas in both cases there were some small changes in path weights in the final models, paths from earlier reading to later intelligence remained significant and model fit was similar, indicating that our primary result was robust to a variety of model-fitting approaches. Illustrations of these two additional models are provided in the online Supporting Information as models S1 and S2, respectively, accompanied by their fit indices.

Third, since the possible effects of reading on intelligence may be contingent on the mean level of reading (a slight advantage over a cotwin whose reading is very poor may be more meaningful than a slight advantage over a cotwin who reads well), we tested whether the results were consistent across the lower, middle, and upper tertiles of the mean reading distribution. Running the model again in these three subsets of the data resulted in very similar results; for example, the largest cross-lagged path, between age 12 reading and age 16 intelligence remained substantial and significant in all three tertiles (path weights .42, .21, and .36 in the lower, middle, and upper tertiles, respectively).

Fourth, we tested the sensitivity of the reading composite by using only teacher-rated reading as the reading variable at all four ages at which it was available (7, 9, 10, and 12 years). The resulting model is illustrated in online Figure S3. Significant associations of reading rating at age 7 were found with intelligence at ages 10 and 12 (path weights .12 and .10, respectively). Thus, restricting the reading measurements to one indicator, consistent across four measurement waves, still resulted in significant links between reading differences and intelligence differences. This analysis provides evidence against possible objections to our results based on measurement variance across time.

Overall, the process of path testing supported our hypothesis about the effects of reading on intelligence: Reading differences measured at several ages were significantly associated with later intelligence differences. Only one significant cross-lagged path emerged between intelligence and reading differences: that between age 9 intelligence differences and age 10 reading differences. Contemporaneous covariance pathways between reading and intelligence were retained for four of the five ages, suggesting that elements of the environment experienced at these ages by one twin of a pair that tend to improve reading also tend to raise intelligence.

**Figure 4:**
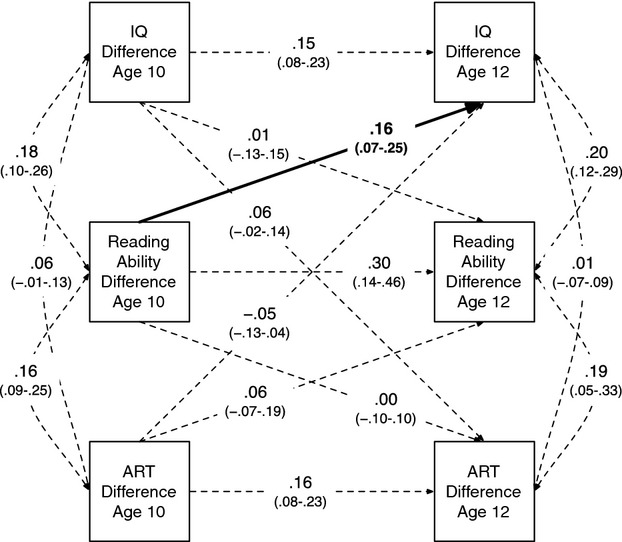
Saturated cross-lagged monozygotic twin difference model of the relations of Author Recognition Test (ART) scores, reading ability, and intelligence between ages 10 and 12. Values are standardized path coefficients with 95% confidence intervals in parentheses. Bold line indicates an association of reading ability with later intelligence where 95% confidence intervals do not cross zero. IQ = general intelligence.

The associations of reading ability with intelligence found in the initial model may, in part, be driven by differences in reading exposure, or alternatively, exposure may have its own, independent links to intelligence beyond those of ability. We thus estimated a second cross-lagged model, including ART scores in addition to the summed reading measures. This model only included data from ages 10 and 12, the two ages at which the ART was administered. Figure4 shows the saturated version of this model. The confidence intervals for the association of earlier ART score differences with later intelligence differences crossed zero, indicating that ART was not significantly associated with subsequent improvements in intelligence.

This was confirmed after a process of dropping nonsignificant paths similar to that described earlier (see Table 3); the resulting model had good fit to the data, χ^2^(7) = 7.80; RMSEA = .006, CFI = .994, TLI = .988, and whereas reading ability differences were positively associated with later intelligence differences, the cross-lagged path from ART score to intelligence was not retained, indicating that reading exposure does not affect intelligence, at least between ages 10 and 12. This model, showing only significant paths, is shown in online Figure S4.

**Table 3:** Changes in Model Fit After Dropping Individual Paths via the Path-Dropping Process for the ART, Reading, and Intelligence Model (Shown in online Figure S2)

Path set	Change in fit after dropping paths		
	ΔAIC	Cumulative no. paths dropped (Δ*df*)	*p*
Within-trait paths	0	0	1
Cross-lagged paths	−5.05	5	.42
Covariance paths	−6.20	7	.35

Note. The saturated model included 15 paths; the final model included 8.

AIC = Akaike information criterion. Reference model AIC = 507.11 on 7 *df*.

As discussed earlier, the assessment of associations between reading and general intelligence may elide differential relations of reading to verbal and nonverbal intelligence. By increasing vocabulary and general knowledge, reading might only improve verbal aspects of intelligence, leaving nonverbal aspects of intelligence unchanged. The association demonstrated in the analysis above, then, may be driven entirely by associations between reading and verbal intelligence. To test this possibility, we estimated a third model in which the intelligence measures were split into their verbal and nonverbal subcomponents.

Again, we tested each individual path from each set; here, however, 12 sets of paths were involved. Using the same rules, we tested the path sets in the following order: within-trait reading paths, within-trait verbal intelligence paths, within-trait nonverbal intelligence paths, between-trait reading–verbal intelligence paths, between-trait reading–nonverbal intelligence paths, between-trait verbal–nonverbal intelligence paths, reading–verbal covariances; reading–nonverbal covariances, and verbal–nonverbal covariances. Fit changes from this process are shown in Table4, while the final model is shown in online Figure S5. The final model fit was excellent, χ^2^(69) = 64.93; RMSEA = .00, CFI > 1.00, TLI > 1.00, and not significantly different from that of the saturated model (*p *=* *.72).

**Table 4:** Changes in Model Fit After Dropping Individual Paths via the Path-Dropping Process for the Model Shown in online Figure S3

Path set	Change in fit after dropping paths		
	ΔAIC	Cumulative no. paths dropped (Δ*df*)	*p*
Reading: within-trait	−3.21	4	.31
VIQ: within-trait	−16.94	13	.77
NVIQ: within-trait	−23.12	19	.73
Reading → VIQ: cross-lagged	−25.33	25	.48
Reading → NVIQ: cross-lagged	−33.53	33	.49
VIQ → reading: cross-lagged	−46.53	43	.62
NVIQ → reading: cross-lagged	−59.42	51	.79
VIQ → NVIQ: cross-lagged	−64.51	60	.64
NVIQ → VIQ: cross-lagged	−73.07	67	.69
VIQ ↔ reading: covariance	−74.83	68	.71
NVIQ ↔ reading: covariance	−73.07	69	.62
VIQ ↔ NVIQ: covariance	−73.07	69	.62

Note. The saturated model included 110 paths; the final model included 41.

AIC = Akaike information criterion; VIQ = verbal intelligence; NVIQ = nonverbal intelligence. Reference model AIC = 10684.39 on 69 *df*.

We found two significant paths between reading differences and nonverbal intelligence differences, specifically, from ages 7 to 9, and from ages 10 to 16; these were direct paths and were not mediated via verbal intelligence. Paths from reading to verbal intelligence differences were also present and overall summed to a larger association of reading with verbal intelligence. The converse cross-lagged paths between intelligence and subsequent reading differences were, unexpectedly, found only for nonverbal intelligence.

### Effect Size

The variance explained by each path in the diagrams included here can be calculated by squaring its path weight. To take one example, reading differences at age 12 in the model shown in Figure3 explain 7% of intelligence differences at age 16 (.26^2^). However, since our measures are of differences, they are likely to include substantial amounts of noise: Measurement error may produce spurious differences. To remove this error variance, we can take an estimate of the reliability of the measures (generally high, since our measures are normed, standardized tests), which indicates the variance expected purely by the reliability of the measure, and subtract it from the observed variance between twins in our sample. Correcting for reliability in this way, the effect size estimates are somewhat larger; to take the above example, the reliability-corrected effect size of age 12 reading differences on age 16 intelligence differences is around 13% of the “signal” variance. It should be noted that the age 12 reading differences themselves are influenced by many previous paths from both reading and intelligence, as illustrated in Figure3.

## Discussion

This article addressed the question of whether intelligence differences may be caused, in part, by nonshared environmentally driven differences in reading, which accumulate across time and transfer to more general cognitive abilities. In a longitudinal analysis of cognitive development in monozygotic twins, assessed in five waves from ages 7 to 16, we found support for this hypothesis. Twins with better earlier reading ability compared to their identical cotwin tended not only to have better reading at subsequent measurements but also higher scores on general intelligence tests. No associations of reading exposure with intelligence were found beyond those of reading ability. We also found that the associations are not restricted to possible effects of reading on the verbal domain—mainly affecting vocabulary and general knowledge—but extend to associations of reading with nonverbal intelligence.

Our cross-lagged monozygotic twin difference design allowed us to rule out many alternative explanations for these links, since both genetic and shared environmental influences, as well as associations between earlier and later intelligence, were controlled. These results, then, support models in which reading is hypothesized to act upon intelligence (e.g., Ritchie & Bates, 2013), and suggest that environmental gains are magnified across time and across traits, causing children within a family to become more different than they otherwise would be (Plomin, 2011).

The results are the strongest support to date for the view of Cunningham and Stanovich (1998) that reading improves verbal intelligence. However, unlike those authors, we did not find associations of reading exposure (measured by the ART) with later intelligence; the only associations in our models were with reading ability. It may be the case that earlier measures of reading exposure—our ART measures were restricted to ages 10 and 12—would have shown significant paths to later intelligence, but alternatively, reading ability may be more important for intellectual development than reading exposure. If the association between reading and verbal ability found here were causal, one potential mechanism may lie in the facilitatory effects of orthography learning on vocabulary, found in studies by Rosenthal and Ehri (2008) and Ricketts, Bishop, and Nation (2009). In these experiments, children who learned new words and nonwords in conjunction with orthography, as opposed to only hearing the nonword spoken aloud, had better recall for the meanings of the words on a later test, indicating an orthographic “scaffolding” effect on vocabulary acquisition.

However, the links between reading and intelligence found in the present study extended beyond verbal intelligence; reading skill was also associated with subsequent increases in nonverbal ability. Mechanisms for this finding are less clear. Speculatively, these mechanisms could be similar to those that may cause education to increase intelligence (e.g., Becker, Lüdtke, Trautwein, Köller, & Baumert, 2012; Brinch & Galloway, 2012): Better reading ability may improve knowledge of specific facts, but it may also allow abstract thinking skills to be gained via the process of taking on the perspectives of fictional or historical characters, or imagining other worlds, times, and scenarios.

Our study also indicated associations of earlier intelligence with later reading, which may at first glance appear consistent with models in which prior semantic knowledge aids reading development (e.g., Seidenberg, 2012). However, these associations were, first, minimal—in the first model, only one path from intelligence differences to reading differences was significant—and second, unexpectedly driven only by nonverbal intelligence, and not via verbal measures that assess semantic knowledge directly, through tasks such as vocabulary tests. The mechanism for this association, then, may be one in which abstract abilities involving the extraction of information from rule-based systems aid the development of reading expertise rather than one in which better semantic representations facilitate reading performance.

Given our monozygotic-difference design, the associations found are purely related to the nonshared environment, and may plausibly—though at this point hypothetically—be thought of as targets for intervention. Significant paths were found emanating from reading differences as early as age 7, which may underscore the importance of early reading intervention (Ehri, 2012; Torgesen, 2004), especially for children at risk for reading disorders. Recent randomized controlled trials have shown that such children's reading difficulties can be to some extent alleviated (e.g., Clarke, Snowling, Truelove, & Hulme, 2010; Hatcher et al., 2006); our findings raise the possibility that such interventions may, over and above their effects on reading, also improve more general cognitive abilities. It should be noted that our study does not gainsay the substantial genetic effects on both reading and intelligence, or their genetic correlation (e.g., Harlaar et al., 2005). It does, however, provide some of the strongest evidence to date for potential nonshared environmental impacts of reading on intelligence, and thus support for the plausibility of effective intervention to raise both variables.

### Limitations and Future Directions

Importantly, the present study has not ascertained the ultimate nonshared environmental mechanisms causing the initial differences in reading that may lead to intelligence gains. There are many candidates for such mechanisms, all of which may independently influence the reading of one twin from a pair. For instance, effective, high-quality teachers, academically focused peer groups, or specific literature encountered by one twin but not by the other may boost the reward value of learning to read or increase the effectiveness or duration of reading practice.

As noted above, our design partials out the large genetic main effects on intelligence and reading. However, a full picture of cognitive development should include these, along with an appreciation of more complex phenomena. For example, genes that favor reading may lead to children choosing (and evoking) more intellectually stimulating environments through processes Scarr and McCartney (1983) termed “niche picking” or “genotype–environment correlation.” This would lead to further improvements in their cognitive ability via positive feedback loops, similar to those shown to exist here for the environment (note that such genetically influenced mechanisms would not explain the intrapair reading differences in our sample, since the twins were genetically identical, but are highly relevant when taking a more general perspective on child development). Moreover, genetically influenced noncognitive traits, in particular motivation (e.g., Almlund, Duckworth, Heckman, & Kautz, 2011), may also underlie improvements in reading and intelligence via a similar process of active niche picking (e.g., Tucker-Drob & Harden, 2012).

In addition to these genetically linked effects on intelligence, it may also be the case that other environmental interventions beyond reading may have intelligence-boosting potential. For instance, it is plausible that a range of activities undertaken at school, from learning mathematics to practicing self-control, or even physical activity, may contribute to cognitive development. The effects of these further intelligence-enhancing skills should be a focus of future research.

Although our results show that a reading advantage by age 7 is associated with improved later intelligence, age 7 is the earliest point at which reading tests were administered in our sample, and we were thus unable to assess whether reading–intelligence links would be present if reading began prior to age 7. This limitation does not affect our interpretation regarding the importance of early reading, since the reading advantage would have to be in place by 7 for this association to be found. Nevertheless, a similar twin study including measures of very early reading progress would be informative in investigating the precise age at which reading begins to have any effects on cognitive development.

We note that even though our twin-differences model has a major advantage over cross-lagged models that use individual scores, it does not—given that it is not a randomized experiment—rule out all potential noncausal interpretations of our data. Although the results are consistent with causal effects of reading, complex processes of parental or teacher influence could cause both improved reading at earlier ages and improved intelligence at later ages, without reading itself doing the causal “work.”

Finally, our method of using intrapair differences as each variable makes within-individual effect sizes difficult to extract from the final model. Now that we have shown potential effects within MZ twin pairs, researchers may wish to estimate more complex models that would shed further light on individual developmental trajectories. Three such possibilities are the person–fixed effects model, outlined by Allison (2009), which could be adapted to control for genetic and environmental confounds; a combined autoregressive–latent curve model (Bollen & Curran, 2004); and an instrumental-variables approach (e.g., Bollen & Noble, 2011). Use of these alternative modeling strategies—which could also be used to test models more specific than the approach employed in the current article—would also avoid the potential limitations and assumptions of the typical cross-lagged model (see, e.g., Finkel, 1995; Rogosa, 1980). We thank three anonymous reviewers for raising these possibilities, which we recommend are explored in future studies of the reading–intelligence link.

### Conclusion

The present study provided compelling evidence that improvements in reading ability, themselves caused purely by the nonshared environment, may result in improvements in both verbal and nonverbal cognitive ability, and may thus be a factor increasing cognitive diversity within families (Plomin, 2011). These associations are present at least as early as age 7, and are not—to the extent we were able to test this possibility—driven by differences in reading exposure. Since reading is a potentially remediable ability, these findings have implications for reading instruction: Early remediation of reading problems might not only aid in the growth of literacy, but may also improve more general cognitive abilities that are of critical importance across the life span.

## Supporting Information

**Table S1.** Pearson Correlation Matrix for Raw (Uncorrected for Age and Sex) Reading Variables, Including Both Twins From Each Pair **Table S2.** Pearson Correlation Matrix for Raw (Uncorrected for Age and Sex) Intelligence Variables, Including Both Twins From Each Pair **Table S3.** Pearson Correlation Matrix for Summed and Age- and Sex-Corrected Reading and Intelligence Measures, Including Both Twins From Each Pair **Table S4.** Pearson Correlation Matrix for Intelligence and Reading Difference Scores **Figure S1.** Model Resulting From Running the Modeling Process in Reverse **Figure S2.** Model Resulting From a Process of Starting With a Base Model (Including Only First-Order Within-Variable Paths, Nonsignificant Paths Not Shown) **Figure S3.** Saturated Sensitivity Analysis Model Including Only Teacher Ratings of Reading and General Intelligence Variables, Showing 95% Confidence Intervals (in Parentheses) Around Each Path Weight **Figure S4.** Reduced Cross-Lagged Monozygotic Twin Difference Model for Author Recognition Test (Reading Exposure), Reading Ability, and General Intelligence Differences (for Saturated Model, see Figure 3 in the Main Document) **Figure S5.** Three-Tier Cross-Lagged Monozygotic Twin Difference Model Including Reading Ability and Verbal and Nonverbal Intelligence Scores
