# Supplementary material for: Does Learning to Read Improve Intelligence? A Longitudinal Multivariate Analysis in Identical Twins From Age 7 to 16
Source: Child Dev. 2014 Jul 24;86(1):23–36. doi: 10.1111/cdev.12272 (PMC4354297; doi:10.1111/cdev.12272)
Supplement: Supplementary file 1 [file cdev0086-0023-sd1.doc]

Supporting Online Materials for

**Does learning to read improve intelligence?**

**A longitudinal multivariate analysis in identical twins from age 7 to 16**

Stuart J. Ritchie, Timothy C. Bates, & Robert Plomin

**Supplementary Tables**

*Table S1*. Pearson correlation matrix for raw (uncorrected for age and sex) reading variables, including both twins from each pair.

|  | 1 | 2 | 3 | 4 | 5 | 6 | 7 | 8 | 9 | 10 | 11 |
| --- | --- | --- | --- | --- | --- | --- | --- | --- | --- | --- | --- |
| 1. TOWRE age 7 | - |  |  |  |  |  |  |  |  |  |  |
| 2. TRR age 7 | .69 | - |  |  |  |  |  |  |  |  |  |
| 3. TRR age 9 | .64 | .63 | - |  |  |  |  |  |  |  |  |
| 4. PIAT age 10 | .49 | .46 | .46 | - |  |  |  |  |  |  |  |
| 5. TRR age 10 | .60 | .61 | .64 | .46 | - |  |  |  |  |  |  |
| 6. PIAT age 12 | .41 | .38 | .43 | .53 | .47 | - |  |  |  |  |  |
| 7. GOAL age 12 | .34 | .33 | .39 | .39 | .39 | .57 | - |  |  |  |  |
| 8. WJ-RF age 12 | .61 | .48 | .55 | .43 | .52 | .43 | .34 | - |  |  |  |
| 9. TOWRE age 12 | .68 | .48 | .55 | .42 | .54 | .41 | .34 | .60 | - |  |  |
| 10. TRR age 12 | .51 | .46 | .56 | .46 | .54 | .44 | .38 | .48 | .38 | - |  |
| 11. Passages age 16 | .31 | .30 | .35 | .38 | .39 | .45 | .41 | .32 | .28 | .38 | - |
| 12. WJ-RF age 16 | .56 | .46 | .56 | .37 | .51 | .39 | .35 | .68 | .50 | .39 | .35 |

Note: All correlations significant at *p* < .001. Shaded blocks indicate correlations of variables summed together for composite measures (see Table S3). Differences between these values and separate correlations for twin 1 and twin 2 from each pair were negligible. TOWRE = Test of Word Reading Efficiency; TRR = Teacher-Rated Reading (UK National Curriculum level); PIAT = Peabody Individual Achievement Test; WJ-RF = Woodcock-Johnson Reading Fluency (Yes/No).

*Table S2.* Pearson correlation matrix for raw (uncorrected for age and sex) intelligence variables, including both twins from each pair.

|  | 1 | 2 | 3 | 4 | 5 | 6 | 7 | 8 | 9 | 10 | 11 | 12 | 13 | 14 | 15 | 16 | 17 |
| --- | --- | --- | --- | --- | --- | --- | --- | --- | --- | --- | --- | --- | --- | --- | --- | --- | --- |
| 1. Vocab. age 7 | - |  |  |  |  |  |  |  |  |  |  |  |  |  |  |  |  |
| 2. Simil. age 7 | .54 | - |  |  |  |  |  |  |  |  |  |  |  |  |  |  |  |
| 3. Pic. Comp. age 7 | .28 | .24 | - |  |  |  |  |  |  |  |  |  |  |  |  |  |  |
| 4. Concept. Group. age 7 | .25 | .27 | .19 | - |  |  |  |  |  |  |  |  |  |  |  |  |  |
| 5. Vocab. age 9 | .28 | .24 | .14 | .10 | - |  |  |  |  |  |  |  |  |  |  |  |  |
| 6. Info. age 9 | .24 | .19 | .16 | .11 | .43 | - |  |  |  |  |  |  |  |  |  |  |  |
| 7. Pic. Comp. age 9 | .19 | .21 | .15 | .18 | .33 | .27 | - |  |  |  |  |  |  |  |  |  |  |
| 8. Raven’s age 9 | .25 | .28 | .18 | .18 | .33 | .30 | .53 | - |  |  |  |  |  |  |  |  |  |
| 9. Vocab. age 10 | .30 | .26 | .18 | .14 | .37 | .28 | .29 | .35 | - |  |  |  |  |  |  |  |  |
| 10. Info. age 10 | .29 | .21 | .14 | .11 | .36 | .42 | .27 | .33 | .48 | - |  |  |  |  |  |  |  |
| 11. Pic. Comp. age 10 | .13 | .09 | .24 | .13 | .21 | .18 | .23 | .34 | .35 | .36 | - |  |  |  |  |  |  |
| 12. Raven’s age 10 | .20 | .20 | .23 | .20 | .29 | .23 | .39 | .52 | .47 | .40 | .44 | - |  |  |  |  |  |
| 13. Vocab. age 12 | .36 | .31 | .20 | .13 | .32 | .23 | .24 | .27 | .40 | .32 | .20 | .32 | - |  |  |  |  |
| 14. Info. age 12 | .33 | .29 | .21 | .12 | .31 | .38 | .25 | .28 | .37 | .48 | .19 | .32 | .43 | - |  |  |  |
| 15. Pic. Comp. age 12 | .16 | .11 | .29 | .11 | .20 | .10 | .22 | .27 | .23 | .20 | .45 | .37 | .29 | .27 | - |  |  |
| 16. Raven’s age 12 | .20 | .20 | .22 | .19 | .21 | .15 | .35 | .41 | .27 | .24 | .27 | .54 | .37 | .36 | .37 | - |  |
| 17. Vocab. age 16 | .33 | .27 | .17 | .13 | .30 | .28 | .21 | .25 | .32 | .32 | .10 | .26 | .36 | .37 | .18 | .24 | - |
| 18. Raven’s age 16 | .24 | .24 | .26 | .18 | .22 | .16 | .30 | .37 | .26 | .21 | .23 | .50 | .30 | .29 | .32 | .52 | .34 |

Note: All correlations significant at *p* < .001. Shaded blocks indicate correlations of variables summed together for composite measures (see Table S3). Differences between these values, and separate correlations for twin 1 and twin 2 from each pair, were negligible. Vocab. = Vocabuary; Simil. = Similarities; Pic. Comp. = Picture Completion; Concept. Group. = Conceptual Grouping; Info. = Information; Raven’s = Raven’s Progressive Matrices.

*Table S3.* Pearson correlation matrix for summed and age- and sex-corrected reading and intelligence measures, including both twins from each pair. Cronbach’s alpha for the reliability of each composite measure (calculated using raw, uncorrected scores) is provided in the rightmost column.

|  | 1 | 2 | 3 | 4 | 5 | 6 | 7 | 8 | 9 |
| --- | --- | --- | --- | --- | --- | --- | --- | --- | --- |
| 1. Reading age 7 | - |  |  |  |  |  |  |  |  |
| 2. IQ age 7 | .43 | - |  |  |  |  |  |  |  |
| 3. Reading age 9 | .67 | .41 | - |  |  |  |  |  |  |
| 4. IQ age 9 | .41 | .39 | .39 | - |  |  |  |  |  |
| 5. Reading age 10 | .64 | .47 | .62 | .53 | - |  |  |  |  |
| 6. IQ age 10 | .46 | .27 | .40 | .34 | .49 | - |  |  |  |
| 7. Reading age 12 | .70 | .47 | .66 | .48 | .76 | .48 | - |  |  |
| 8. IQ age 12 | .51 | .30 | .44 | .32 | .51 | .61 | .59 | - |  |
| 9. Reading age 16 | .53 | .40 | .53 | .36 | .60 | .41 | .73 | .44 | - |
| 10. IQ age 16 | .34 | .41 | .40 | .43 | .50 | .34 | .53 | .36 | .49 |

Note: All correlations significant at *p* < .001. Differences between these values and separate correlations for twin 1 and twin 2 from each pair were negligible. Reliability not calculated for Reading age 9 as only one measure was taken. IQ = general intelligence.

*Table S4*. Pearson correlation matrix for intelligence and reading difference scores.

|  | 1 | 2 | 3 | 4 | 5 | 6 | 7 | 8 | 9 | 10 | 11 |
| --- | --- | --- | --- | --- | --- | --- | --- | --- | --- | --- | --- |
| 1. Reading difference age 7 | - |  |  |  |  |  |  |  |  |  |  |
| 2. IQ difference age 7 | .17*** | - |  |  |  |  |  |  |  |  |  |
| 3. Reading difference age 9 | .20*** | .09* | - |  |  |  |  |  |  |  |  |
| 4. IQ difference age 9 | .11** | .04 | .06 | - |  |  |  |  |  |  |  |
| 5. Reading difference age 10 | .26*** | .08* | .17*** | .15*** | - |  |  |  |  |  |  |
| 6. ART difference age 10 | .05 | .03 | .06 | .08* | .16*** | - |  |  |  |  |  |
| 7. IQ difference age 10 | .16*** | .03 | .09* | .15*** | .18*** | .05 | - |  |  |  |  |
| 8. Reading difference age 12 | .31*** | .06 | .06 | .10 | .32*** | .13 | .01 | - |  |  |  |
| 9. ART difference age 12 | .08 | -.03 | .04 | .04 | .04 | .16*** | .06 | .20** | - |  |  |
| 10. IQ difference age 12 | .12** | .08* | .04 | .06 | .17*** | -.02 | .18*** | .26*** | .01 | - |  |
| 11. Reading difference age 16 | .08 | .04 | .13* | .01 | .09 | .13** | .00 | .11 | .07 | .00 | - |
| 12. IQ difference age 16 | .07 | .08* | .10* | .06 | .14** | .11* | .06 | .20** | .08 | .12** | .18*** |

Note: ‘Reading ability’ refers to summed ability variables; ART = Author Recognition Test; IQ = general intelligence. * = *p* < .05; ** = *p* < .01; *** = *p* < .001.

**Supplementary Figures**

*Figure S1.* Model resulting from running the modeling process in reverse (see Results section, main document). Values are standardized path weights with 95% confidence intervals in brackets; non-significant paths not shown. Fit indices: χ^2^(27) = 27.84; CFI = .99; TLI = 1.00.

*Figure S2.* Model resulting from a process of starting with a base model (including only first-order within-variable paths, Non-significant paths not shown. Fit indices: *χ*^2^(24) = 14.81; RMSEA = .00; TLI > 1; CFI > 1.

*Figure S3.* Saturated sensitivity analysis model including only teacher ratings of reading and general intelligence variables, showing 95% confidence intervals (in parenthesis) around each path weight. Bold lines indicate associations of reading rating differences with later intelligence differences where 95% CIs do not cross zero.

*Figure S4*. Reduced cross-lagged monozygotic twin difference model for Author Recognition Test (reading exposure), reading ability, and general intelligence differences (for saturated model, see Figure 3 in the main document). Values are standardized path weights; 95% confidence intervals are provided in parentheses. Bold path indicates a significant relationship between earlier reading ability differences and later intelligence differences. Fit indices: *χ*^2^(7) = 7.80; RMSEA = .006; CFI = .994; TLI = .988.

*Figure S5*. 3-tier cross-lagged monozygotic twin difference model including reading ability and verbal and non-verbal intelligence scores. Bold lines highlight associations of earlier reading with later verbal and non-verbal intelligence. 95% confidence intervals are given in parenthesis for each path weight. VIQ = verbal intelligence; NVIQ = non-verbal intelligence. Fit indices: *χ*^2^(69) = 64.93, RMSEA = .00; CFI > 1.00; TLI > 1.00.
